# *Mycobacterium tuberculosis* clinical isolates of the Beijing and East-African Indian lineage induce fundamentally different host responses in mice compared to H37Rv

**DOI:** 10.1038/s41598-019-56300-6

**Published:** 2019-12-27

**Authors:** Bas C. Mourik, Jurriaan E. M. de Steenwinkel, Gerjo J. de Knegt, Ruth Huizinga, Annelies Verbon, Tom H. M. Ottenhoff, Dick van Soolingen, Pieter J. M. Leenen

**Affiliations:** 1 Department Medical Microbiology & Infectious Diseases, Erasmus University Medical Center, Rotterdam, The Netherlands; 2 Department of Immunology, Erasmus University Medical Center, Rotterdam, The Netherlands; 3 Department of Internal Medicine, Erasmus University Medical Center, Rotterdam, The Netherlands; 4 Department of Infectious Diseases, Leiden University Medical Center, Leiden, The Netherlands; 5 National Tuberculosis Reference Laboratory, National Institute of Public Health and the Environment (RIVM), Bilthoven, The Netherlands

**Keywords:** Tuberculosis, Molecular medicine, Antibodies, Infection, Acute inflammation

## Abstract

Substantial differences exist in virulence among *Mycobacterium tuberculosis* strains in preclinical TB models. In this study we show how virulence affects host responses in mice during the first four weeks of infection with a mycobacterial strain belonging to the Beijing, East-African-Indian or Euro-American lineage. BALB/c mice were infected with clinical isolates of the Beijing-1585 strain or the East-African Indian (EAI)-1627 strain and host responses were compared to mice infected with the non-clinical H37Rv strain of the Euro-American lineage. We found that H37Rv induced a ‘classical’ T-cell influx with high IFN-γ levels, while Beijing-1585 and EAI-1627 induced an influx of B-cells into the lungs together with elevated pulmonary IL-4 protein levels. Myeloid cells in the lungs appeared functionally impaired upon infection with Beijing-1585 and EAI-1627 with reduced iNOS and IL-12 expression levels compared to H37Rv infection. This impairment might be related to significantly reduced expression in the bone marrow of IFN-γ, TNF-α and IFN-β in mice infected with Beijing-1585 and EAI-1627, which could be detected from the third day post infection onwards. Our findings suggest that increased virulence of two clinical isolates compared to H37Rv is associated with a fundamentally different systemic immune response, which already can be detected early during infection.

## Introduction

Tuberculosis (TB) is a leading cause of death among infectious diseases worldwide and claimed more victims in 2017 than HIV and malaria combined^1^. While global efforts have resulted in a steady decline in TB-related deaths over the years, new threats present themselves in the form of drug resistance and the emergence of more virulent *Mycobacterium tuberculosis* (Mtb) genotypes^1–3^.

Most TB cases are caused by modern lineage mycobacterial strains of the East Asian/Beijing lineage, the East-African Indian lineage and the Euro-American lineage^2,4,5^. Mycobacterial strains belonging to the Beijing genotype particularly have shown an aggressive global spread over the last century and have been associated with higher rates of treatment failure and disease relapse compared to other genotypes^6–12^. An important reason for the clinical impact of the Beijing genotypes, seems to be their increased capacity to acquire drug resistance^13^. A less well-defined characteristic concerns their hypervirulence^14–16^.

In preclinical TB models, virulent Beijing strains cause higher mycobacterial loads, more lung damage and earlier mortality compared to strains from other lineages^15,17,18^. Mechanistic studies have suggested that Beijing strains have enhanced capacity to inhibit protective immunity in the lungs through induction of higher levels of type-I interferons, leading to lower IL-12 and TNF-α levels and reduced T-cell activation^19,20^. Increased Beijing virulence also has been attributed to bacterial phenolic glycolipid (PGL), which suppresses the production of IL-12, IL-6 and TNF-α by host immune cells^21,22^. Lastly, Beijing strains may induce a stronger regulatory T-cell response compared to other strains, thereby down-regulating protective immunity^17,23^.

In the current study we evaluate the host response during acute infection against the virulent Beijing-1585 strain. This strain has previously demonstrated similar infection and mortality kinetics as other virulent Beijing strains^18,24^. Furthermore, Beijing-1585 was found associated with drug resistance and treatment failure^18,25^. We compare Beijing-1585 with the East-African/Indian (EAI)-1627 strain, that displays similar virulence as Beijing-1585 in our model^18^, and with the less virulent H37Rv strain belonging to the Euro-American lineage^26^.

Previous studies in our BALB/c mouse TB model showed that mice infected with H37Rv reach maximal mycobacterial loads and start developing progressive pneumonia 28 days post infection (dpi). Next, they enter a phase of chronic infection and become moribund between 22 and 38 weeks post infection^26^. In contrast, mice infected with Beijing-1585 or EAI-1627 reach peak infection at 14 dpi with histopathological signs of pneumonia comparable to H37Rv at 28 dpi and rapidly become moribund between three to five weeks post infection if left untreated^26,27^. In this study we aim to identify the differences in underlying host responses that might contribute to this marked difference in virulence.

## Results

### Infection with Beijing-1585 or EAI-1627 results in higher mycobacterial loads compared to H37Rv

**Figure 1:**
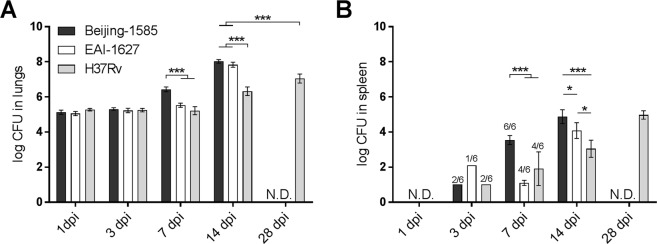
Mycobacterial loads in lungs and spleen. (**A**) Mycobacterial loads in the lungs after intratracheal infection with Beijing-1585 (black bars), EAI-1627 (open bars) or H37Rv (grey bars). After 14 days Beijing-1585- and EAI-1627-infected mice rapidly become moribund, therefore no later analyses for these strains are possible. (**B**) Mycobacterial loads in the spleen. Three mice were used for each group at 1 dpi and 6 mice for each group at the remaining time points. The numbers above the bars indicate the number of mice out of n = 6 in total with positive cultures. Inoculum sizes were 1.0.10^5^ CFU for Beijing, 1.3.10^5^ CFU for EAI and 1.8.10^5^ CFU for H37Rv. *p < 0.05 **p < 0.01 ***p < 0.001 after Bonferroni correction.

After intratracheal infection of BALB/c mice with *Mycobacterium tuberculosis* (Mtb), we found no significant differences in mycobacterial load between Beijing-1585, EAI-1627 and H37Rv at 1 day post infection (dpi) and 3 dpi, indicating that all groups received a similar inoculum of mycobacteria (Fig. 1A). At 7 dpi, mice infected with Beijing-1585 had significantly higher mycobacterial loads and at 14 dpi, Beijing-1585 and EAI-1627 caused almost 2 log higher loads than H37Rv. Mycobacterial loads for H37Rv at peak infection (28 dpi) were still one log value lower than those observed for Beijing-1585 and EAI-1627 at 14 dpi. These findings are in agreement with our and others’ previous studies monitoring mycobacterial loads for Beijing strains and H37Rv^15,28,29^.

To assess whether the higher mycobacterial loads in the lungs caused by Beijing-1585 and EAI-1627 were associated with more rapid dissemination to other organs, we determined mycobacterial loads in the spleen (Fig. 1B). No significant differences in culture positivity rate were found between strains, but mycobacterial loads in the spleens of mice infected with Beijing-1585 were higher at 7 dpi and 14 dpi compared to other groups.

### Beijing-1585 and EAI-1627 induce lung influx of B-cells, while H37Rv induces T-cell influx

**Figure 2:**
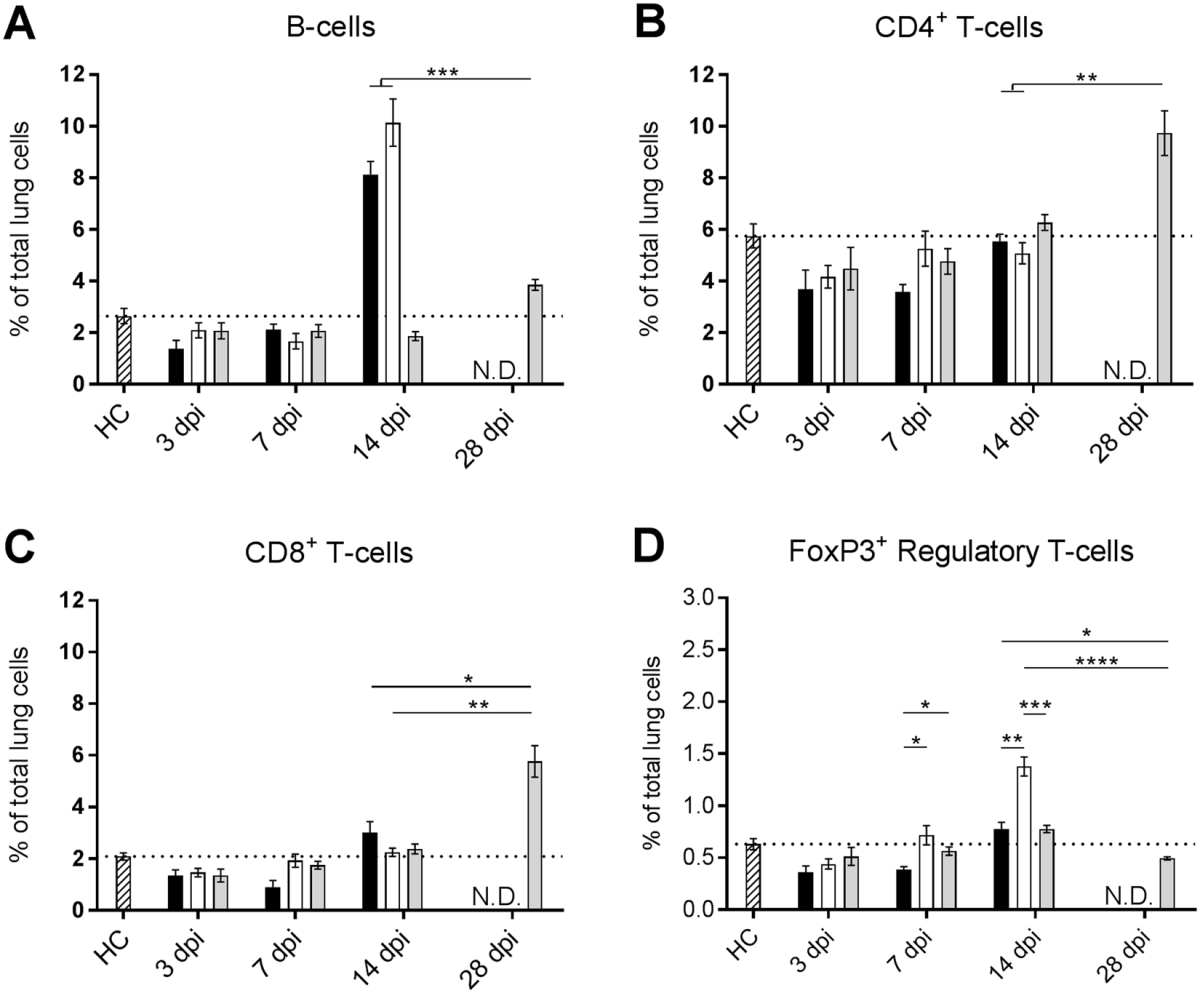
Lymphoid cell populations in the lungs of mice infected with different Mtb strains. Lymphoid cells were determined in the lungs of mice infected with Beijing-1585 (black bars), EAI-1627 (open bars) or H37Rv (grey bars) and compared to uninfected control mice (HC, striped bars). Gating strategies are shown in Fig. S5. (**A**) B-cells, identified based on scatterplot and high level CD45R expression, are significantly higher in number for Beijing-1585- and EAI-1627-infected mice at 14 dpi compared with H37Rv at 14 and 28 dpi. (**B**,**C**) Only H37Rv infection induces an increase in both CD4^+^ and CD8^+^ T-cells at 28 dpi. (**D**) Despite the increase in T-cells caused by H37Rv infection at 28 dpi, Foxp3^+^ regulatory T-cells are significantly lower compared to Beijing-1585- and EAI-1627-infected mice at 14 dpi. Gating strategies were similar as described previously^51^. N = 6 mice per group per time point, *p < 0.05, **p < 0.01, ***p < 0.001 after Bonferroni correction. Data are shown as % of total lung single cell suspension.

To explore whether the distinct *in vivo* mycobacterial growth profiles in our model correlated with differences in adaptive immune responses, we evaluated the numbers of B- and T-cells recruited to the lungs by the three different strains. Most notably, Beijing-1585 and EAI-1627 induced a strong influx of CD45R^+^ cells, identifying B-lymphocytes, at 14 dpi, which was not observed for H37Rv at either 14 dpi or 28 dpi (Fig. 2A). In contrast, H37Rv induced the recruitment of CD4^+^ and CD8^+^ T-cells at 28 dpi, which in turn was not observed upon infection with the clinical strains (Fig. 2B,C). Despite the marked T-cell increase in the H37Rv group at 28 dpi, Foxp3^+^ regulatory T-cell percentages of total lung single cell suspension remained lower compared to Beijing-1585 and EAI-1627 at 14 dpi (Fig. 2D). The latter strains show a marked difference in FoxP3^+^ Treg at 14 dpi, where only mice infected with EAI-1627 show an elevated Treg level above background.

**Figure 3:**
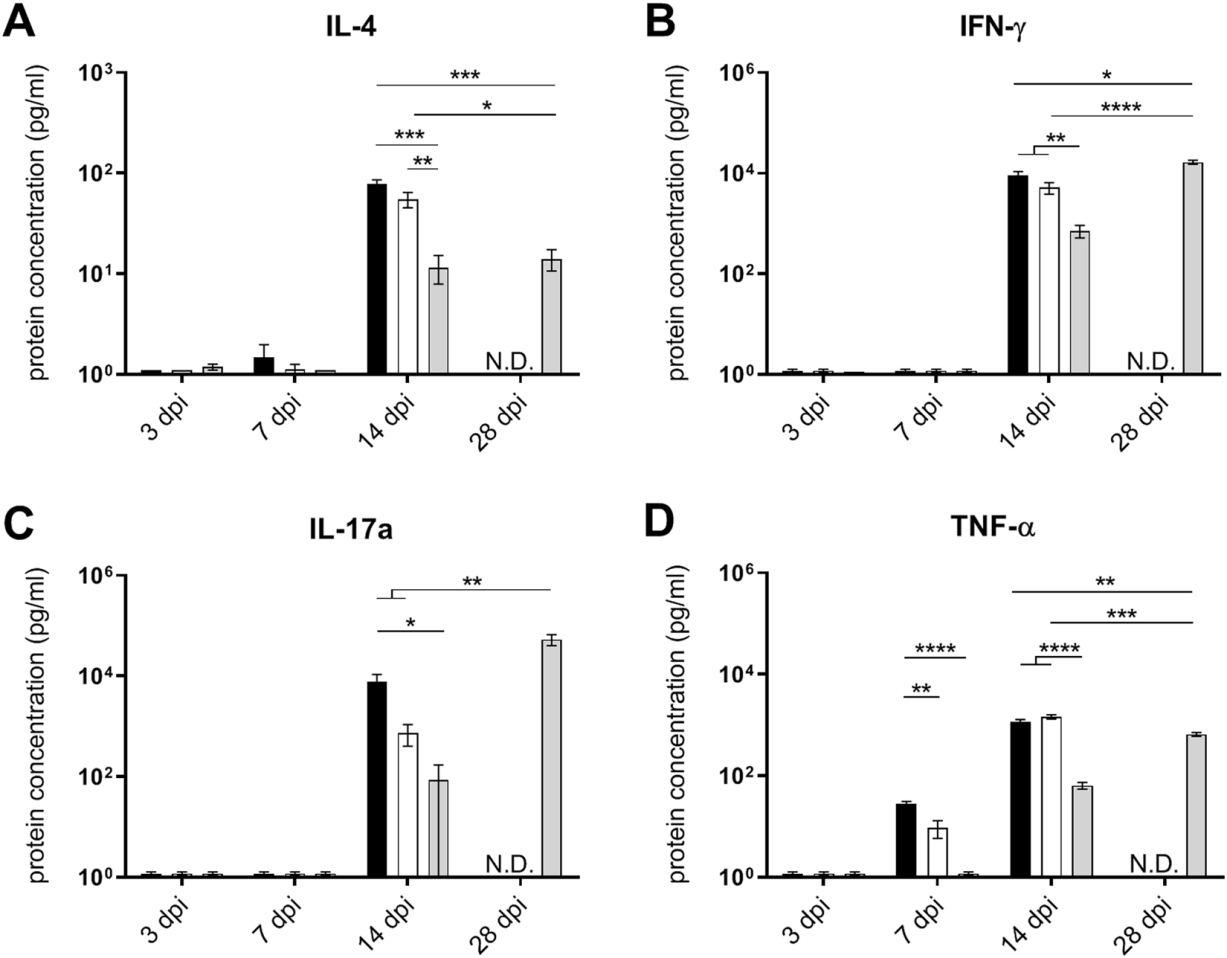
Cytokine protein levels in the lungs of mice infected with different Mtb strains. Protein levels were determined in lung tissue homogenates of mice infected with Beijing-1585 (black bars), EAI-1627 (open bars) or H37Rv (grey bars). (**A**) IL-4 levels are 4–5 fold higher for Beijing-1585 and EAI-1627 at 14 dpi compared to H37Rv at 14 dpi or 28 dpi. (**B**) IFN-γ levels are elevated for Beijing-1585 and EAI-1627 at 14 dpi, but are 2-fold lower compared to H37Rv at 28 dpi. (**C**) Beijing-1585 and EAI-1627 induced circa 7-fold lower levels of IL-17a at 14 dpi compared to H37Rv at 28 dpi. (**D**) TNF-α levels are circa 2-fold higher for Beijing-1585 and EAI-1627 at 14 dpi compared to H37Rv at 28 dpi. N = 5 mice per group per time point for Beijing-1585 and H37Rv and n = 4 mice for EAI, *p < 0.05, **p < 0.01, ***p < 0.001, ****p < 0.0001 after Bonferroni correction.

The associated cytokine protein levels in the lungs for each time point and genotype strain are shown in Fig. 3. In accordance with the increase in B-cells, Beijing-1585- and EAI-1627-infected mice showed elevated protein levels of IL-4 at 14 dpi. These were 4–5 fold higher than IL-4 levels observed for H37Rv at 14 or 28 dpi (Fig. 3A). Although Beijing-1585 and EAI-1627 also caused elevated protein levels of IFN-γ and IL-17a at 14 dpi, these remained 2-fold and 6-fold lower, respectively, compared to the H37Rv group at 28 dpi (Fig. 3B,C).

The TNF-α protein levels in the lungs closely correlated with strain-dependent differences in mycobacterial loads over time. At 7 dpi, TNF-α levels were significantly induced only in mice infected with Beijing-1585- and EAI-1627, which were almost 2-fold higher at 14 dpi compared to H37Rv at 28 dpi (Fig. 3D). This is in line with the role of TNF-α as general inflammation marker. In support of this, the inflammation marker IL-6 showed similar kinetics as TNF-α over time (Fig. S1). IL-10 and IL-23 levels were also measured in the lung homogenates but were below the limit of detection of our assay (data not shown). Quantitative PCR measurements of IFN-γ, IL-17a TNF-α, IL-6 in the lungs were performed with outcomes comparable to those at protein level as shown in Fig. 3 (Fig. S2). IL-10 expression levels were above the lower limit of detection but did not show strain-specific differences (Fig. S2).

### Beijing-1585 and EAI-1627 induce a qualitatively impaired myeloid response compared to H37Rv

**Figure 4:**
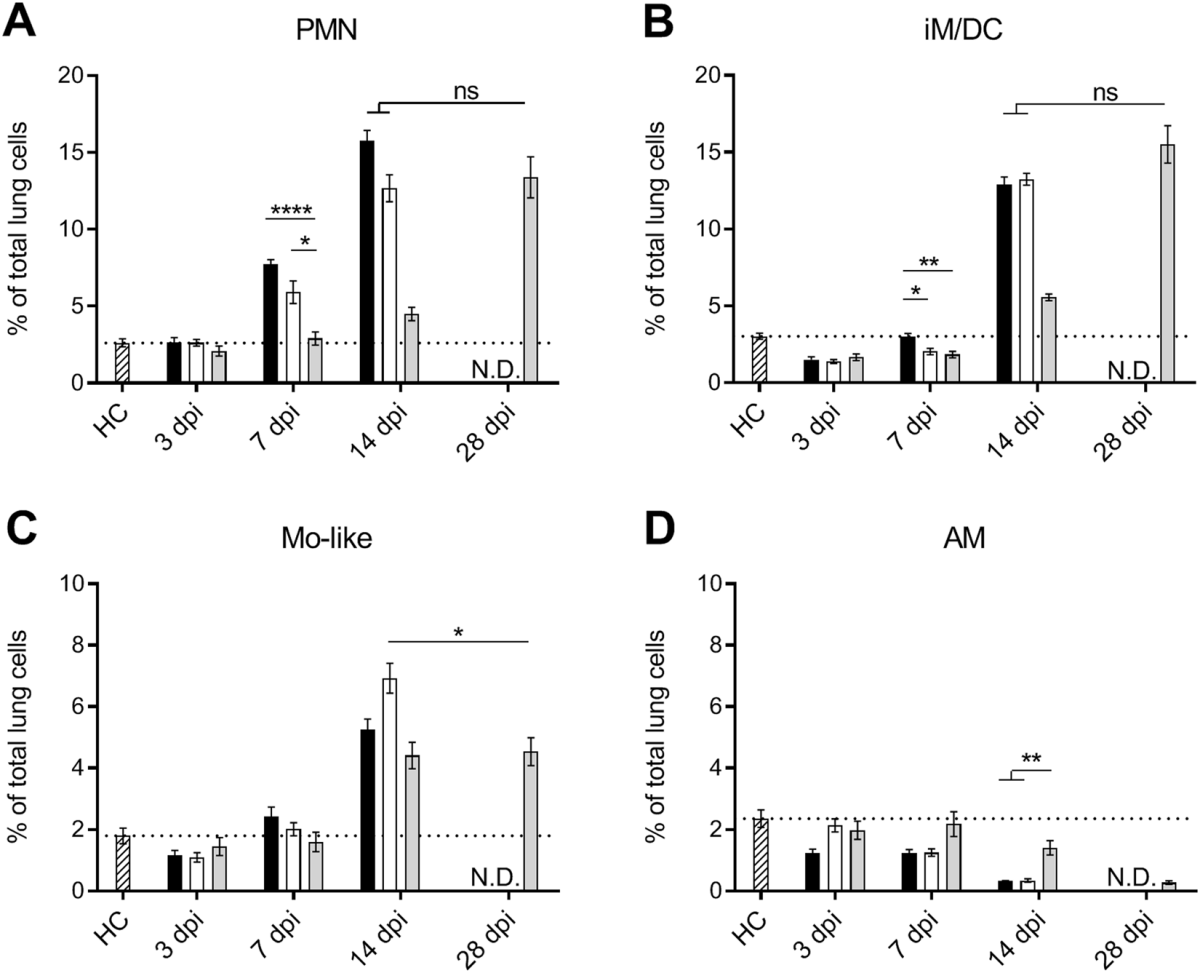
Myeloid cell populations in the lungs of mice infected with different Mtb strains. CD11b^+^ cells were classified as PMN (CD11b^+^Ly6G^high^), iM/DC (CD11b^+^Ly6C^int^CD11c^high^), monocyte-like cells (Mo-like) (CD11b^+^Ly6C^high^CD11c^low^) and alveolar macrophages (AM) (CD11b^int^CD11c^high^Siglec-F^+^) in the lungs of mice infected with Beijing-1585 (black bars), EAI-1627 (open bars) or H37Rv (grey bars) compared to uninfected control mice (HC, striped bars). Gating strategies are shown in Fig. S5. (**A**) PMN cells showed a more rapid increase for Beijng-1585 and EAI-1627 compared to H37Rv. (**B**) iM/DC showed kinetics comparable to PMN for the different groups. (**C**) Monocyte-like cells are only higher in the EAI-1627 group at 14 dpi compared to the H37Rv group at 28 dpi. (**D**) Lung alveolar macrophages are lower in the Beijing-1585 and EAI-1627 group compared to the H37Rv group at 14 dpi. N = 6 mice per group per time point, *p < 0.05, **p < 0.01, ***p < 0.001 after Bonferroni correction.

The observed differences in lymphoid cell responses and cytokine levels raised the question whether CD11b^+^ myeloid cell influxes in the lungs might also vary between mice infected with different Mtb strains. Lung polymorphonuclear granulocyte (PMN) percentages were increased in the Beijing-1585 and EAI-1627 group compared to H37Rv at 7 dpi and 14 dpi (Fig. 4A), which was in line with the elevated mycobacterial loads and inflammation markers TNF-α and IL-6 at these time points. However, at 28 dpi the PMN frequency in the H37Rv group was comparable with that in the Beijing-1585 group and EAI-1627 group at 14 dpi.

Inflammatory macrophage/dendritic cell (iM/DC; CD11b^+^Ly6C^int^CD11c^high^) influx showed a similar profile as PMN (Fig. 4B). Monocyte-like cells (CD11b^+^Ly6C^high^CD11c^low^) were present to a lesser extent than PMN and iM/DC, and were only higher in the EAI-1627 group at 14 dpi compared to the H37Rv group at 28 dpi (Fig. 4C). Alveolar macrophages (AM) were reduced over time in all groups, associated with inflammatory cell influx, but most prominently at 14 dpi in the Beijing-1585 and EAI-1627 groups compared to the H37Rv group (Fig. 4D). We also evaluated lung eosinophils in each group since these cells are known IL-4 producers, but levels of these cells were not elevated in the Beijing-1585 and EAI-1627 groups compared to the H37Rv group at any time point evaluated (Fig. S3).

**Figure 5:**
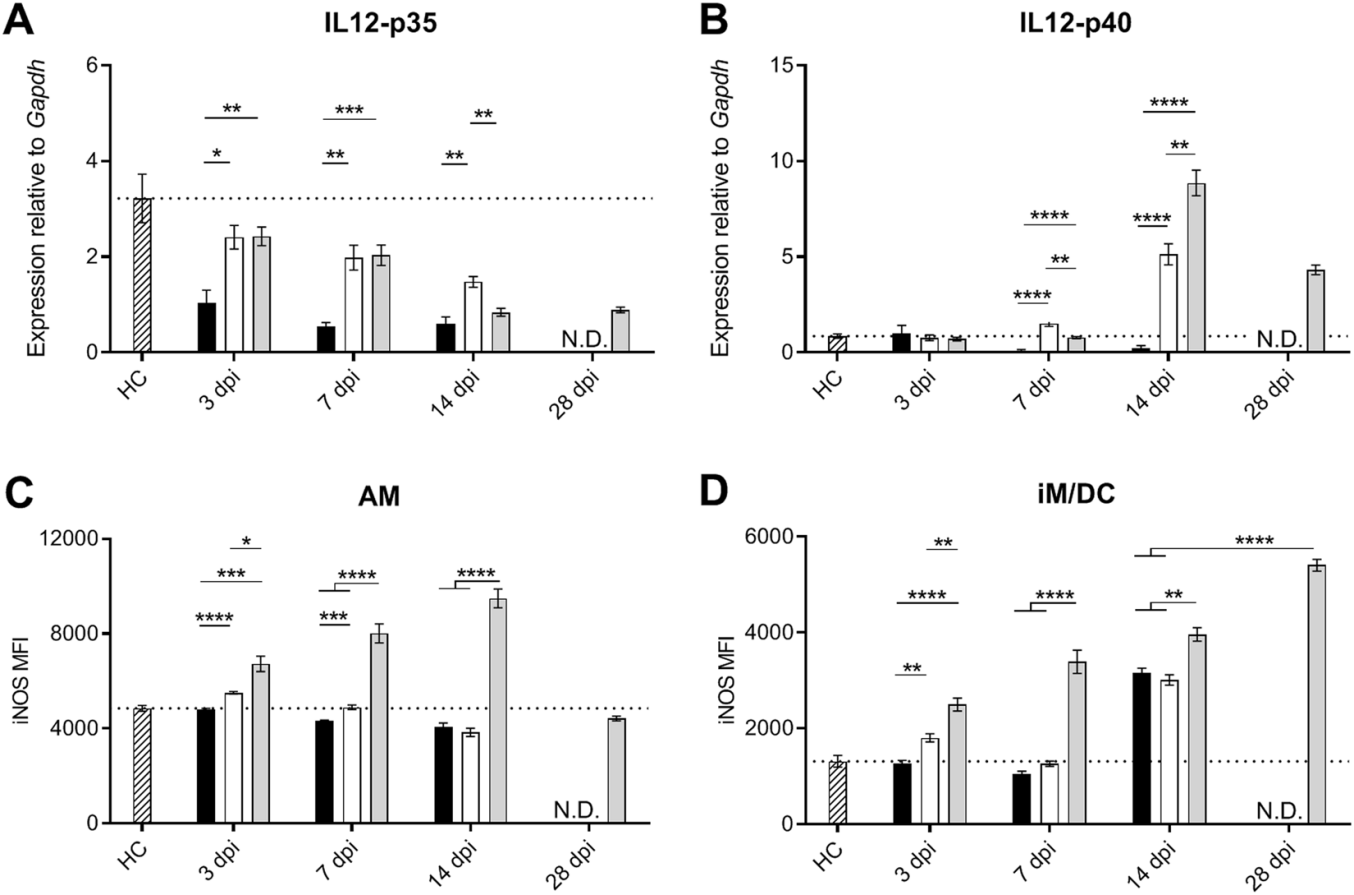
IL-12- and myeloid iNOS expression in the lungs of Mtb-infected mice. IL-12 mRNA expression in total lung homogenate as determined by qPCR (**A**,**B**) and iNOS expression assessed by flow cytometry in (**C**) alveolar macrophages (AM) and (**D**) inflammatory macrophages and dendritic cells (iM/DC) in the lungs of mice infected with Beijing-1585 (black bars), EAI-1627 (open bars) or H37Rv (grey bars) are compared to uninfected control mice (HC, striped bars). (**A**) IL-12p35 expression levels are lower in the Beijing-1585 group compared to the EAI-1627 and H37Rv group. (**B**) IL-12p40 expression is induced in EAI-1627- and H37Rv-infected mice, and reached its peak for both strains at 14 dpi with higher expression in the H37Rv group. Beijing-1585 does not induce any notable expression of either IL-12p35 or IL-12p40 at all time points evaluated. (**C**) Only AM in the lungs of H37Rv-infected mice at 3,7 and 14 dpi show iNOS expression levels higher than observed in uninfected control mice. (**D**) also iM/DC in H37Rv-infected mice show higher iNOS expression than iM/DC in the lungs of mice from the Beijing-1585 or EAI-1627 group at all time points evaluated. N = 6 mice per group per time point, *p < 0.05, **p < 0.01, ***p < 0.001 after Bonferroni correction. For experiments depicted in this figure, iM/DC were defined as CD11b^high^CD11c^high^MHC-II^+^ cells and AM were defined as CD11b^int^CD11c^+^F4/80^+^CD200R^+^ cells (gating strategies: Fig. S4A), based on a distinct panel of antibodies. Population frequencies through this gating were highly comparable to those in Fig. 4 (Fig. S4B).

The AM and iM/DC are important cellular sources of IL-12 in the lungs^30^, which is essential for initiation of T-cell responses^31^. Therefore, we measured the expression of IL-12p35 and IL-12p40. Most notably, Beijing-1585 caused the strongest down-regulation of IL-12p35 in the lungs compared to uninfected control mice and did not induce any IL-12p40 expression at all time points evaluated (Fig. 5A,B).

To investigate induction of putative bactericidal activity^30,32^ we measured the expression of inducible nitric oxide synthase (iNOS), typically inducible by IFN-γ and TNF-α, in both infiltrating iM/DC and the lung-resident AM at each time point. The AM from mice infected with the clinical strains essentially failed to up-regulate iNOS at any point during the course of infection (Fig. 5C). In contrast, iNOS expression was already significantly higher in AM from H37Rv-infected mice at 3 dpi and its expression continued to increase up to 14 dpi.

The iM/DC in lungs of mice infected with Beijing-1585 or EAI-1627 also showed significantly lower iNOS expression compared to H37Rv-infected mice at all time points (Fig. 5D). At 14 dpi, when IFN-γ and TNF-α levels were high in the lungs of mice from the Beijing-1585 and EAI-1627 group (Fig. 3C), iNOS expression by iM/DC was increased accordingly. Nevertheless, iNOS expression by iM/DC in H37Rv-infected mice was significantly higher at 14 dpi despite lower levels of IFN-γ and TNF-α in the lungs compared to Beijing-1585- and EAI-1627-infected mice. iNOS expression by iM/DC in H37Rv-infected mice increased even further at 28 dpi.

### Infection with Beijing-1585 induces less inflammatory cytokines in bone marrow compared to H37Rv

**Figure 6:**
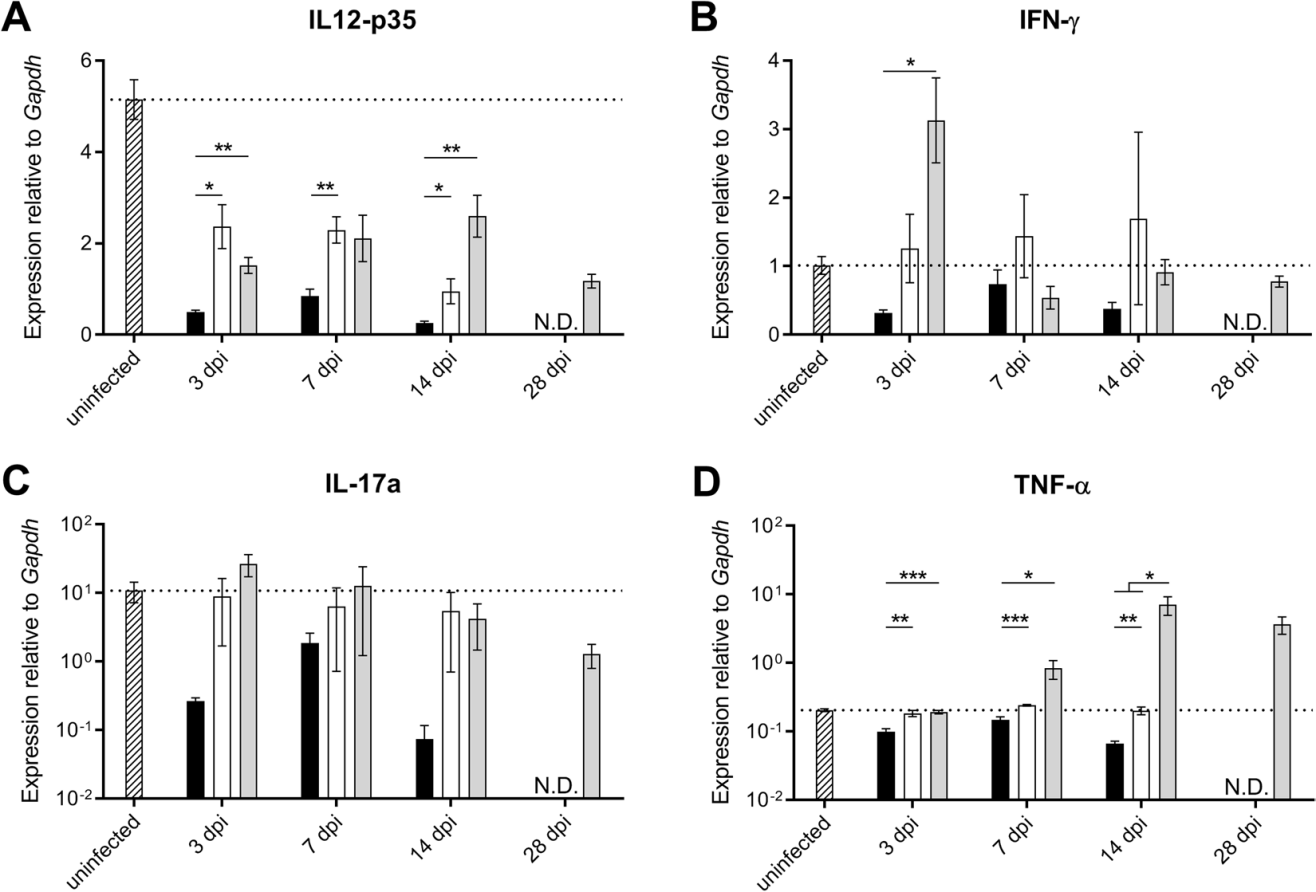
Cytokine mRNA expression levels in bone marrow of mice infected with different Mtb strains. Expression levels of target cytokine mRNA are shown relative to *Gapdh* in the bone marrow of mice infected with Beijing-1585 (black bars), EAI-1627 (open bars) or H37Rv (grey bars). (**A**) IL-12p35 expression at 3 dpi and 7 dpi was lower in the Beijing-1585 group compared to H37Rv. (**B**) IFN-γ expression was lower for Beijing-1585 at 3 dpi compared to H37Rv. (**C**) IL-17a levels were markedly lower in the Beijing-1585 group compared to the H37Rv group (mean of 0.26 vs. 26.7), but without statistical significance (p = 0.06 after Bonferroni correction) due to the high spread in the H37Rv group (5.3–71.8). (**D**) TNF-α levels were higher in the bone marrow of H37Rv-infected mice compared to Beijing-1585-infected mice at all time points evaluated. EAI-1627 consistently showed intermediate results between Beijing-1585 and H37Rv for all cytokines. N = 6 mice per group per time point, *p < 0.05, **p < 0.01, ***p < 0.001, ****p < 0.0001 after Bonferroni correction.

The differential induction of iNOS in infiltrating iM/DC might be caused by distinct local inflammatory or inhibitory conditions. Alternatively, cells might be differently primed at an earlier developmental stage. Therefore, we determined cytokine mRNA expression in the bone marrow in the course of infection. Similar to the lungs, IL-12p35 mRNA expression in the bone marrow was down-regulated most effectively by Beijing-1585 infection compared to uninfected mice at all time points evaluated (Fig. 6A). Interestingly, Beijing-1585 infection also showed a lack of induction, or even reduced expression of inflammatory cytokines IFN-γ, IL-17a and TNF-α compared to H37Rv as early as 3 dpi (Fig. 6B–D). Especially for TNF-α, expression levels differed markedly between bone marrow cells from Beijing-1585- and H37Rv-infected mice over time with a decreased expression for Beijing-1585 at 14 dpi compared to 3 dpi, as opposed to a 34-fold increase for H37Rv. Measurement results for the EAI-1627 group consistently were intermediate between those for the Beijing-1585 and H37Rv groups.

### Induction of type 1 IFN signature genes in the lungs of infected mice essentially correlates with expression of IFN-β

**Figure 7:**
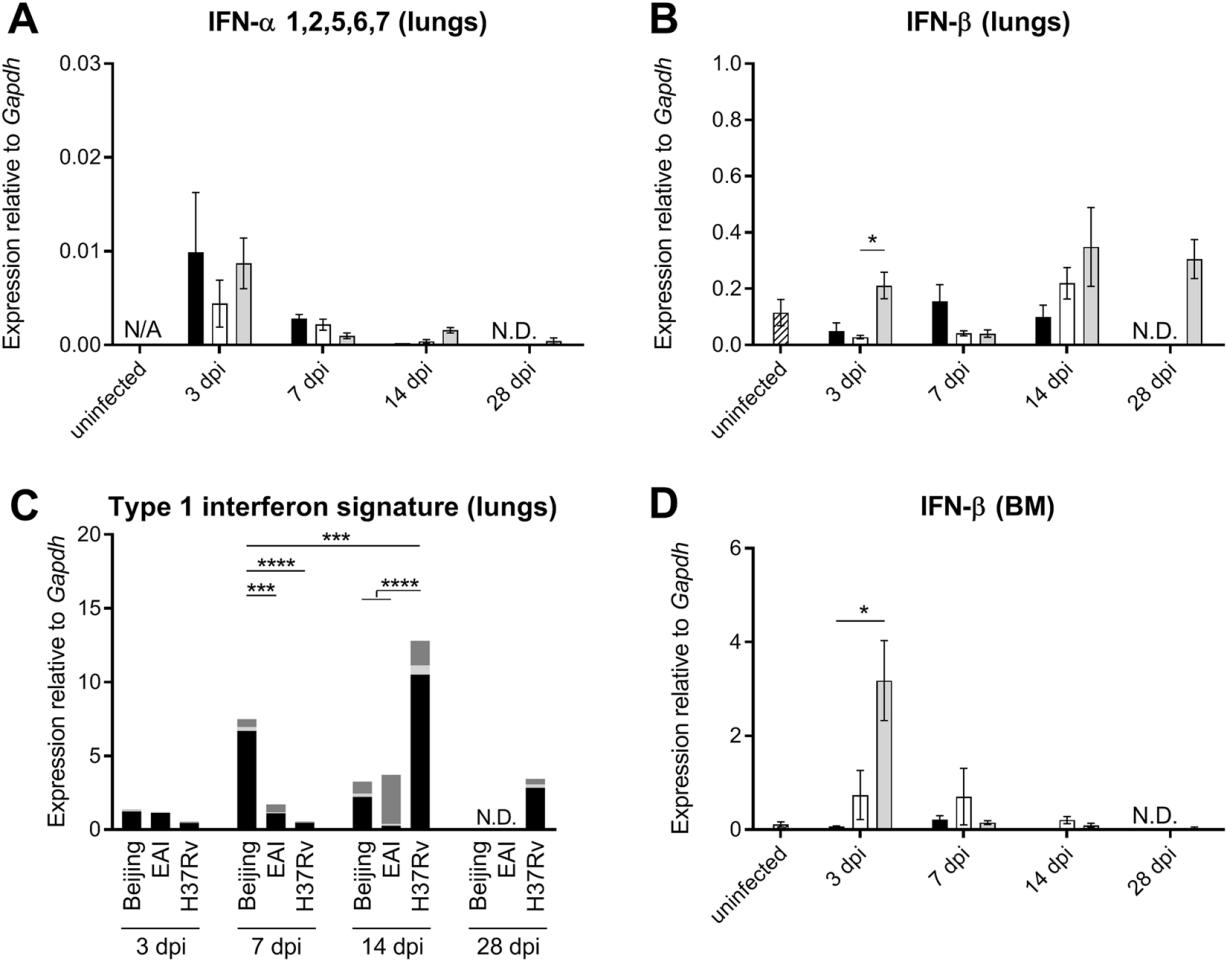
Expression of IFN-α, IFN-β and type 1 interferon-inducible genes in lung and bone marrow during infection with different Mtb strains. (**A**) Combined expression of IFN-α 1,2,5,6,7 mRNA in lung homogenate did not show significant differences between groups. No expression could be detected in uninfected mice (N/A). (**B**) Expression of IFN-β mRNA in lung homogenate was higher in the lungs of H37Rv-infected mice compared to EAI-1627-infected mice at 3 dpi. Other differences did not reach significance. (**C**) Combined expression in the lungs of type 1 interferon-inducible genes *Mx1* (black), *IFI44* (light grey) and *CCL2* (dark grey), represented in a type 1 interferon signature, showed the highest level in the Beijing-1585 group at 7 dpi and in the H37Rv group at 14 dpi. For individual expression levels see Fig. S6. (**D**) Expression of IFN-β in the bone marrow is higher in the H37Rv group compared to Beijing-1585 at 3 dpi. N = 6 mice per group per time point, *p < 0.05, **p < 0.01, ***p < 0.001 after Bonferroni correction. Two-way, repeated measure ANOVA followed by Bonferroni correction was used to calculate significance for **C**.

Since mycobacterial virulence has been associated with increased lung induction of type 1 interferons, we tested the mRNA expression of IFN-α genes (subtypes 1, 2, 5, 6 and 7) in the lungs in a similar approach as originally described by Manca *et al*.^20^. We found only a limited expression of the tested IFN-α genes at 3 dpi for all strains, which decreased upon progressing infection and showed no significant inter-strain differences (Fig. 7A).

IFN-α and IFN-β share the ability to bind to, and signal via the IFN-α/β receptor, therefore we evaluated IFN-β mRNA expression in the lungs. IFN-β expression in the H37Rv group was significantly higher than that in the EAI-1627 group at 3 dpi, and higher than that in the Beijing-1585 group, but without statistical significance (Fig. 7B).

Type I interferons comprise several more subtypes than those for which we could test expression by qPCR and their expression is often transient. Therefore we decided to test the type 1 interferon response, represented by expression of *Mx1*, *IFI44* and *CCL2*, which are known type 1 interferon-inducible genes^33–35^. Expression levels of such genes can be combined into a type 1 interferon signature, which provides an indication of type 1 interferon responsiveness in a tissue^36^. Our type 1 interferon signature showed different kinetics between Beijing-1585 and H37Rv, with EAI-1627 again showing intermediate results (Fig. 7C, see Fig. S5 for individual graphs). Most notably, at 7 dpi the Beijing-1585 group showed the strongest induction of type 1 interferon-inducible genes in the lungs, while H37Rv-infected mice showed a higher peak induction at 14 dpi. The observed kinetics for the type 1 interferon-inducible genes at 7 dpi and 14 dpi closely matched the trends observed for IFN-β expression at these time points, and not IFN-α, except for the absence of a type 1 interferon signature in the lungs of mice infected with H37Rv at 3 dpi. This suggests that in this model IFN-β is more relevant for the induction of type 1 IFN-regulated genes during acute infection than the tested IFN-α subtypes.

To compare the findings on IFN-β in the lungs at 3 dpi with differential systemic effects observed in the bone marrow, we measured IFN-β mRNA expression in the bone marrow. This showed a significantly increased expression of IFN-β in the H37Rv group compared to the Beijing-1585 group at 3 dpi (Fig. 7D). As such, the profile showed similarities to the expression of IFN-γ in bone marrow (Fig. 6C).

## Discussion

We found that infection with the *Mycobacterium tuberculosis* Beijing-1585 strain or EAI-1627 strain is characterized by an influx of B-cells in the lungs and higher pulmonary IL-4 protein levels compared to a T cell-dominated response to the less virulent H37Rv strain. Beijing- or EAI-strain infection is also associated with recruitment of myeloid cells that appear functionally impaired with low IL-12 and iNOS expression levels. In addition, especially Beijing-1585 infection is associated with a reduced expression of inflammatory cytokines in the bone marrow compared to H37Rv-infected mice, as early as from 3 dpi onwards, suggesting disrupted priming of developing myeloid cells in favor of the mycobacteria.

Could the increased B-cell influx upon infection with clinical *Mycobacterium tuberculosis* strains contribute to the observed difference in virulence in our model? In chronic TB patients a protective role for B-cells and antibodies was recently demonstrated^37,38^. A study in non-human primates showed that B-cell depletion during acute infection resulted in lower levels of inflammation, but higher bacterial burdens^39^. We found that higher percentages of B-cells were associated with higher levels of inflammation, as expressed by inflammation markers TNF-α and IL-6, but also with higher bacterial burdens. In a mouse TB model of acute TB, B-cells were protective as they reduced neutrophilia by limiting IL-17 responses^40^. We did find lower IL-17 protein levels in the lungs of Beijing-1585- and EAI-1627-infected mice compared to H37Rv at peak infection, but we observed a similar influx of PMN into the lungs. More recently it was shown that IL-17 and IL-17-producing innate lymphoid cells were protective during acute Mtb infection^41–43^. Our findings of higher IL-17 levels in the lungs of H37Rv-infected mice compared to Beijing-1585 and EAI-1627 are in accordance with this notion. Nevertheless, the exact role of B cells and potential involvement of IL-17 in this axis remains to be established.

Beijing-1585 and EAI-1627 infection elicited significant IL-4 protein levels in the lungs, thus matching the observed increase in B-cells. The presence of IL-4 was previously shown to exert a pathogenic effect during TB by diverting the role of TNF-α from myeloid cell activator to tissue damage mediator^44^. This is in line with observations by others that virulent Beijing strains induce higher IL-4 mRNA expression levels in the lungs of mice at 14 dpi compared to non-virulent Beijing strains^24^, and *in vitro* studies showing that Beijing-HN878 preferentially induced IL-4 expression in human peripheral blood mononuclear cells compared to the CDC1551 strain^45^. Together with our observations, this adds to the support for a host-detrimental role of IL-4 and B-cell responses during acute infection in which IL-4 stimulates macrophages to express a non-bactericidal, alternatively activated phenotype. However, differential exposure of myeloid cells to IL-4 may not be decisive in pathogenesis, since similar mortality, bacterial burden, histopathology and iNOS expression were observed after infection of wildtype or myeloid-specific IL-4Ralpha knockout mice with H37Rv or HN878^46^. The source of IL-4 during acute infection remains obscure as no notable T-cell responses were observed for Beijing-1585 and EAI-1627. Also eosinophil numbers, as another known source of IL-4 in the lungs, were not elevated in our study. Given the recently demonstrated protective role of IL-17- producing group 3 ILC in TB^43^, it might be interesting to study the role of IL-4 producing group 2 ILC during acute infection with Beijing or EAI strains.

Beijing-1585 induced lower lung IL-12p35 and IL-12p40 mRNA expression levels compared to H37Rv, which is in agreement with previous studies^20,21,45,47^. This might contribute to reduced T-cell responses given the role of IL-12 in this axis^31,48^. However, the differences in IL-12 expression between EAI-1627 and H37Rv were less pronounced and EAI-1627 infection still resulted in a B-cell response, making this a less likely explanation for the noticeable difference in lymphocyte response. iNOS-expression levels in AM and iM/DC were reduced both in mice infected with Beijing-1585 and EAI-1627 compared to H37Rv, consistent with previous studies^15,24,49^. Low iNOS expression was previously shown to be associated with low expression levels of IFN-γ and TNF-α mRNA^32^. Remarkably, we observed that lower iNOS expression by iM/DC was accompanied with higher IFN-γ and TNF-α protein levels in the lungs of Beijing-1585- and EAI-1627-infected mice compared to H37Rv at 14 dpi. This low level iNOS expression in AM and iM/DC in the lungs despite high local IFN-γ protein levels could be due to inhibition or prevention of iNOS induction at the site of infection. Alternatively, iNOS expression might be affected by differential priming of myeloid cells at an earlier stage of development.

In support of our hypothesis that differential priming might play a role we found that the expression of inflammatory cytokines such as IFN-γ and TNF-α in the bone marrow differs significantly between mice infected with different bacterial strains. Interestingly, this occurs already in the early stage of infection before widespread bacterial dissemination. While not conclusive, this suggests that differential bone marrow priming of myeloid cells might be an important shaping factor early during infection with *Mycobacterium tuberculosis*.

Next to differential expression of IFN-γ in the bone marrow, we found significant differences in type 1 interferon expression and responses in both bone marrow and lung between Beijing-1585 and H37Rv. Previous studies have associated virulent Beijing-HN878 infection with elevated IFN-α mRNA expression in the lungs at 28 dpi in a low-dose BALB/c infection model^19,20^. We were unable to reproduce this preferential increase in IFN-α mRNA upon infection with the virulent Beijing strain in the lungs, which could be due to using a high-dose infection model and/or measurements at different time points. However, other parameters such as bacterial load kinetics and host responses in H37Rv infection in our model show strong similarities to low-dose (10^2^ CFU) infection studies^17,50^ suggesting that strain-dependent virulence plays a dominant role in pathogenicity over bacterial load. Also, in our subsequent analysis of both generic type 1 interferon-inducible gene expression and pulmonary IFN-β expression we found generally lower expression levels after infection with Beijing-1585 and EAI-1627 compared to H37Rv, thus questioning the direct association of *Mycobacterium tuberculosis* virulence with type 1 interferon activity during acute infection.

Taken together, we show in a mouse TB model that infection with highly virulent clinical Beijing- and EAI-strains is associated with influx of B-cells and elevated IL-4 levels in the lungs, while less virulent H37Rv bacteria induce T-cell influx and higher IFN-γ and IL-17a levels at peak infection. Induction of iNOS is hampered in lung-infiltrating myeloid cells, especially in Beijing-infected mice. A significantly reduced expression in the bone marrow of IFN-γ, TNF-α and IFN-β, early in infection with virulent clinical Mtb strains, might contribute to this poor responsiveness of myeloid cells recruited in the lungs.

## Materials and Methods

### Mycobacterial strains

We used the *Mycobacterium tuberculosis* H37Rv strain (ATCC 27294) belonging to the Euro-American lineage and two strains isolated from patients in Vietnam in 2002, Beijing-1585, and EAI-1627, as representatives for their respective lineage based on genotyping results^27^.

### Mice and infection

Female specific pathogen-free BALB/c mice aged 10–11 weeks and weighing 22–24 grams (Charles River, Les Oncins, France) were infected by intratracheal instillation under general anesthesia as described previously^25^. Inoculum sizes were confirmed by plating and were 1.0.10^5^ colony forming units (CFU) for Beijing-1585, 1.3.10^5^ CFU for EAI-1627 and 1.8.10^5^ CFU for H37Rv. Mice infected with Beijing-1585 or EAI-1627 rapidly become moribund between 3–5 weeks^18^, therefore mycobacterial loads and other parameters for these two clinical strains were measured up to the peak of infection at 14 dpi, while measurements on H37Rv-infected animals were continued up to peak of infection at 28 dpi.

### Ethics statement

All protocols were approved by the Erasmus MC institutional animal ethics committee (DEC number 117-12-13, EMC-number 3005) and adhered to the rules laid down in the Dutch Animal Experimentation Act and the EU Animal Directive 201/63/EU. All methods were performed in accordance with the relevant guidelines and regulations. The Beijing-1585 and EAI-1627 mycobacterial strains are part of the sample collection of the National Tuberculosis Reference Laboratory, National Institute of Public Health and the Environment (RIVM, Bilthoven, the Netherlands).

### Determination of mycobacterial load

Lungs and spleens were removed aseptically and homogenized in 2 mL PBS using the gentleMACS Octo Dissociator (Miltenyi Biotec BV, Leiden, the Netherlands) according to the manufacturer’s protocol. From each tissue homogenate 10-fold serial dilutions were made in PBS. Next, 200 µL aliquots were plated on 7H10 agar culture plates supplemented with 10% OADC. Plates were incubated for up to 42 days at 37 °C and 5% CO_2_ before colonies were counted.

### Flow cytometry

The flow cytometry protocol, fluorescent antibody panels, real-time quantitative PCR and cytokine assessments were essentially as described previously^51^. Briefly, lung single-cell suspensions were fixed for 30 min in fix/perm solution (Ebioscience, Vienna, AT) to eliminate live bacteria prior to flow cytometry analysis. Next, cells were washed and incubated for 10 min in permeabilization buffer (Ebioscience) and then incubated for 30 min with different mAb mixes as described Supplementary Table 1. After staining, the cells were washed again, resuspended in PBS/BSA/azide buffer and measured on a FACS Canto II flow cytometer (BD Biosciences, Breda, NL).

### Real-time quantitative PCR

RNA from mouse lung homogenate was purified and processed as described previously^51^. Primer sequences and manufacturers are listed in Supplementary Table 2.

### Cytokine protein levels

To determine cytokine levels in the lungs, lung homogenate was placed in a low-adhesion tube (USA Scientific, Orlando, FL, USA) and centrifuged at 10.000 × g for 5 min. The supernatant was collected, placed in an Eppendorf tube with a 0.45 μm filter (Corning BV Life Sciences, Amsterdam, NL) and centrifuged again at 10.000 × g for 5 min to filter out all mycobacteria. IFN-γ, TNF-α, IL-4, IL-6, IL-17a, IL-23 and IL-10 concentrations were measured in the filtrate using a Luminex assay according to manufacturer’s instructions (Merck Millipore, Amsterdam, NL).

### Data analysis and statistics

Flow cytometry data were analyzed using Flowjo 7.6.5. Analyses were done and graphs were made using PRISM GraphPad 7. All data are expressed as mean ± SEM. Student’s t-test, followed by Bonferroni correction for multiple comparisons where applicable, was used to calculate significance, except for Fig. 7B. Here we used two-way, repeated measure ANOVA. P-values less than 0.05 were considered statistically significant.

## Supplementary information

Supplementary information 
