# Supplementary material for: Mycobacterium tuberculosis clinical isolates of the Beijing and East-African Indian lineage induce fundamentally different host responses in mice compared to H37Rv
Source: Sci Rep. 2019 Dec 27;9:19922. doi: 10.1038/s41598-019-56300-6 (PMC6934500; doi:10.1038/s41598-019-56300-6)
Supplement: Supplementary file 1 — Supplementary information [file 41598_2019_56300_MOESM1_ESM.docx]

***Mycobacterium tuberculosis* clinical isolates of the Beijing and East-African Indian lineage induce fundamentally different host responses in mice compared to H37Rv.**

B.C. Mourik, J.E.M. de Steenwinkel, G.J. de Knegt, R. Huizinga, A. Verbon, T.H.M Ottenhoff, D. van Soolingen, P.J.M. Leenen

| **Supplementary Table 1: Fluorescent antibody panels** | | | | | | |
| --- | --- | --- | --- | --- | --- | --- |
|  | **Panel 1** |  |  | **Panel 2** |  |  |
| **Marker** | **Ab** | **Clone** | **Source** | **Ab** | **Clone** | **Source** |
| FITC | CD4 | L3T4 | BD biosciences | CD45R | RA3-6B2 | BD biosciences |
| PE | FoxP3 | FJK-16s | E-Bioscience | Siglec-F | E50-2440 | BD biosciences |
| PerCP Cy5.5 | Ly-6C | ER-MP20 | E-Bioscience | Ly-6C | ER-MP20 | E-Bioscience |
| APC | CD3 | 145-2CN | BD biosciences | CD11b | M1/70 | BD biosciences |
| Pe Cy7 | CD11b | M1/70 | E-Bioscience | CD11c | N418 | E-Bioscience |
| APC Cy7 | CD25 | PC61 | Biolegend | MHC-II | M5/114.15.2 | E-Bioscience |
| PB | CD8 | 53-6.7 | E-Bioscience | CD45 | 30-F11 | Biolegend |
|  | **Panel 3** |  |  | **Panel 4** |  |  |
| **Marker** | **Ab** | **Clone** | **Source** | **Ab** | **Clone** | **Source** |
| FITC | CD68 | FA-11 | Biolegend | iNOS | 6/iNOS | BD biosciences |
| PE | PDL1 | MIH5 | E-Bioscience | CD200R | OX110 | E-Bioscience |
| PerCP Cy5.5 | Ly-6C | ER-MP20 | E-Bioscience | F4/80 | BM8 | E-Bioscience |
| APC | CD117 | ACK2 | E-Bioscience | CD86 | B7-2,GL-1 | E-Bioscience |
| Pe Cy7 | CD11c | N418 | E-Bioscience | CD11c | N418 | E-Bioscience |
| APC Cy7 | CD11b | M1/70 | E-Bioscience | MHC-II | M5/114.15.2 | E-Bioscience |
| PB | Ly-6G | 1A8 | Biolegend | CD11b | M1/70 | E-Bioscience |

| **Supplementary Table 2: Primer sequences and probe numbers used for real-time quantitative PCR** | | | | |  |
| --- | --- | --- | --- | --- | --- |
|  | **Forward primer** | **Reverse primer** | | **Probe*** | **Ref** |
| GAPDH | AGCTTGTCATCAACGGGAAG | TTTGATGTTAGTGGGGTCTCG | | 9 | ^1^ |
| IFN-γ | GCAAAAGGATGGTGACATGA | TTCAAGACTTCAAAGAGTCTGAGG | | 21 | ^1^ |
| TNF-α | CCACGTCGTAGCAAACCAC | TTTGAGATCCATGCCGTTG | | 25 | ^1^ |
| IL-17a | TTTTCAGCAAGGAATGTGGA | TTCATTGTGGAGGGCAGAC | | 34 | ^1^ |
| IL-12p35 | GAGACTTCTTCCACAACAAGAGG | CAGGGTCATCATCAAAGACG | | 27 | ^1^ |
| IL-12p40 | TGGACTGGACTCCCGATG | CATCTTCTTCAGGCGTGTCA | | 80 | ^1^ |
| IL-10 | GCTCCTAGAGCTGCGGACT | TGTTGTCCAGCTGGTCCTTT | | 41 | ^1^ |
| Mx1 | TTCAAGGATCACTCATACTTCAGC | GGGAGGTGAGCTCCTCAGT | | 53 | ^2^ |
| IFN-α1,6,5 | Mm03030145_gH** | | ThermoFisher Scientific | | |
| IFN-α2 | Mm00833961_s1 | | ThermoFisher Scientific | | |
| IFN-α7 | Mm02525960_s1 | | ThermoFisher Scientific | | |
| IFN-β1 | Mm00439552_s1 | | ThermoFisher Scientific | | |
| IFI44 | Mm00505670_m1 | | ThermoFisher Scientific | | |
| CCL2 | Mm00441242_m1 | | ThermoFisher Scientific | | |
| * Universal probe library (Roche), ** ThermoFisher scientific Assay ID | | | | | |

|  |
| --- |
| **Supplementary figure 1: IL-6 protein levels in lung homogenate of Mtb-infected mice**  IL-6 protein levels were determined in lung tissue homogenates of mice infected with Beijing-1585 (black dots), EAI-1627 (open circles) or H37Rv (grey squares). Kinetics were similar to those observed for TNF-α as shown in **Fig. 3D**. * p < 0.05, ** p < 0.01, **** p < 0.0001 after Bonferroni correction. |

|  |
| --- |
| **Supplementary figure 2: mRNA expression of relevant cytokines in the lungs of Mtb-infected mice**  mRNA expression levels relative to *Gapdh* in the lungs of mice infected with Beijing-1585 (black dots), EAI-1627 (open circles) or H37Rv (grey squares). N=6 mice per group per time point, * p < 0.05, ** p < 0.01, **** p < 0.0001 after Bonferroni correction. |

|  |
| --- |
| **Supplementary figure 3: Eosinophils in the lungs of Mtb-infected mice**  Eosinophils in the lungs of mice infected with Beijing-1585 (black bars), EAI-1627 (open bars) or H37Rv (grey bars). ** p < 0.01 after Bonferroni correction. Gating strategies were similar as described previously and Eosinophils were identified based on their high expression of CD11b, Siglec-F and autofluorescence in the APC channel ^3^. |

| 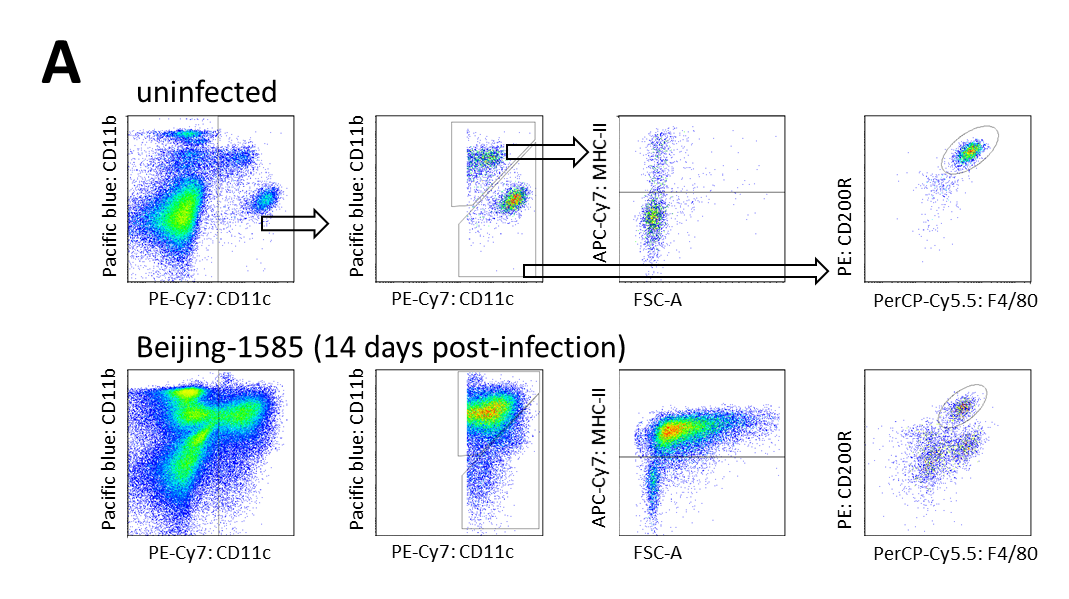 |
| --- |
| **** |
| **Supplementary figure 4: Gating strategies to identify different myeloid cell types and assess iNOS expression in each**  **A**) Gating strategies to identify iM/DC (I) and AM (II) in lung homogenate. iM/DC were separated from other cell populations based on their expression of CD11c, CD11b and MHC-II, while AM were separated based on their high CD11c expression and additional expression of CD200R and F4/80.  **B**) Population size determination of AM and iM/DC through this alternative fluorescent antibody marker panel yielded highly similar results as shown in **Figure 4B/D** in the manuscript, indicating that the same cell populations are identified. |


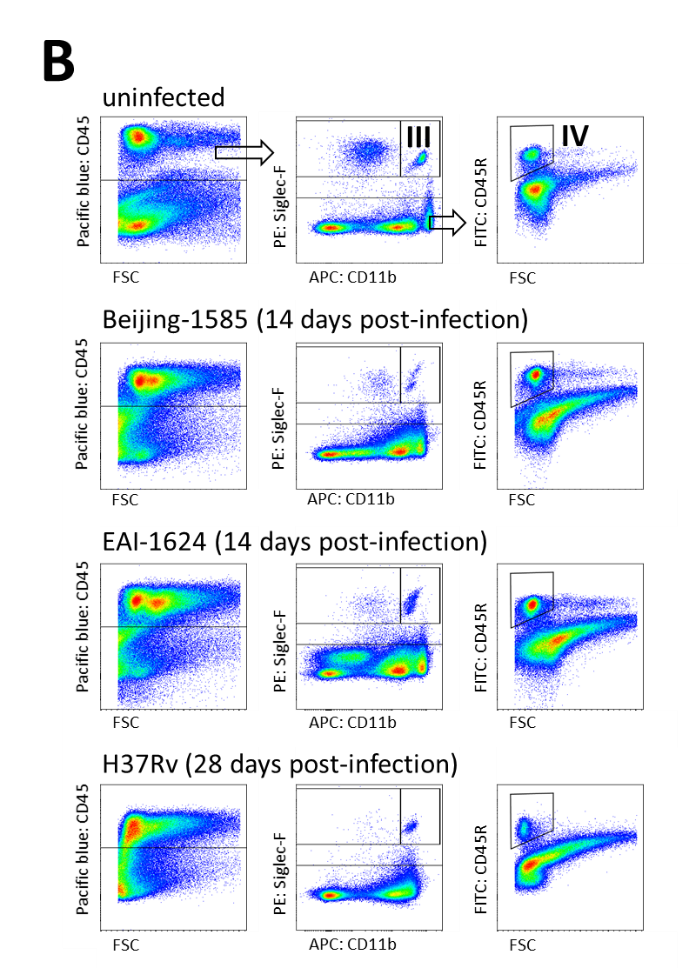

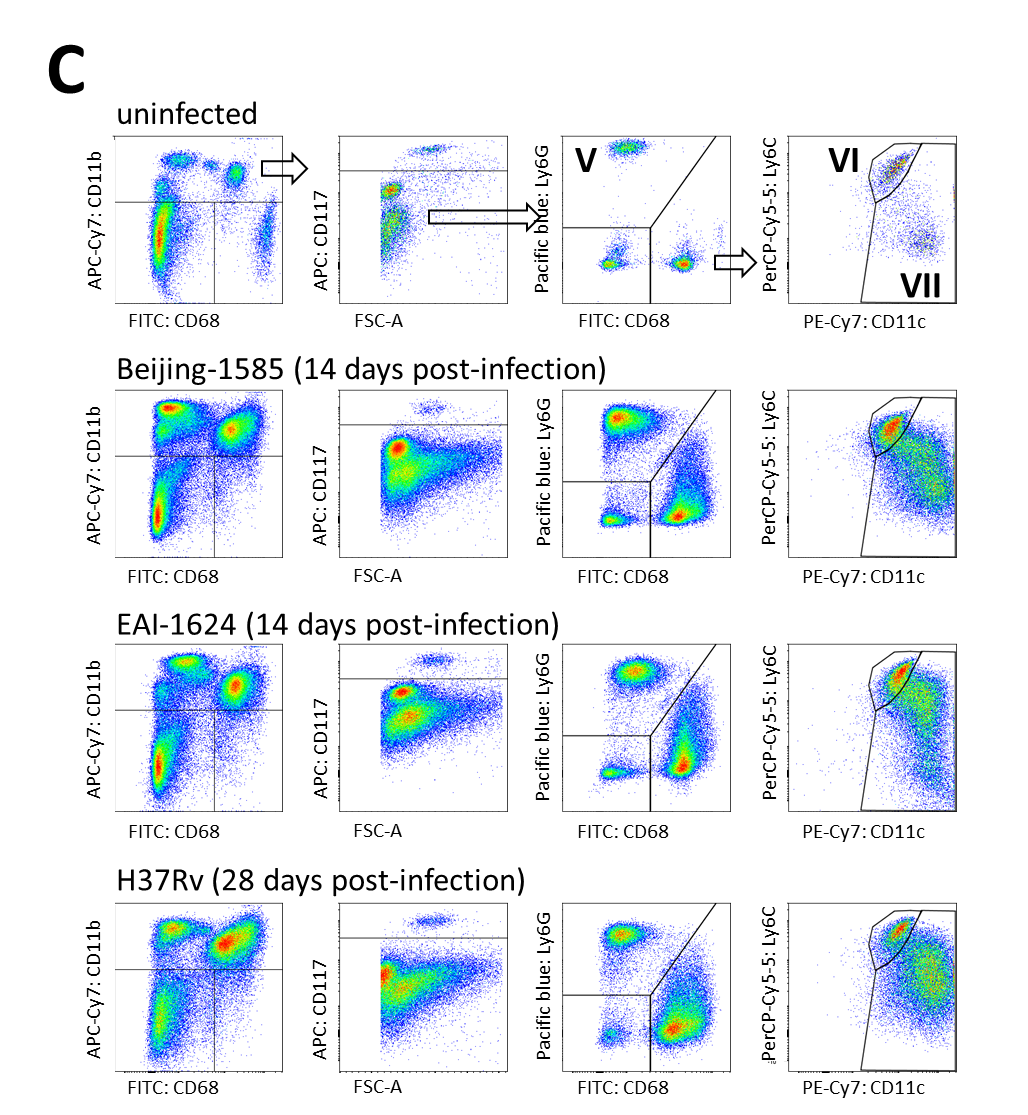

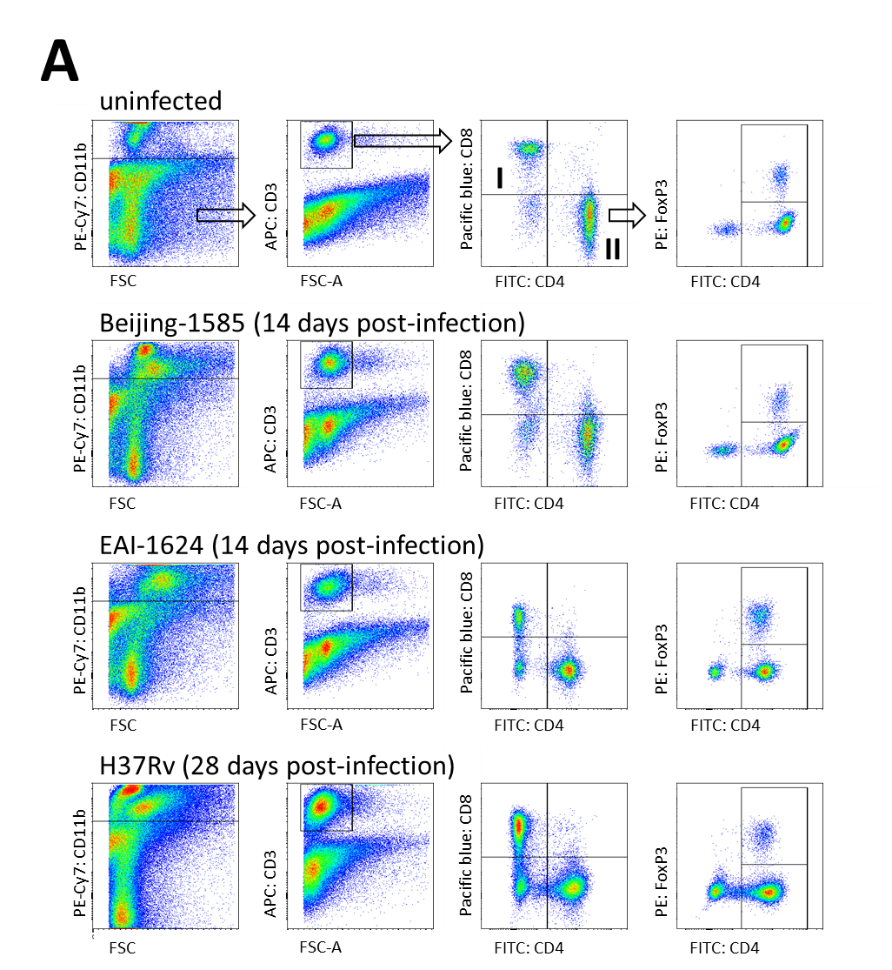


**Supplementary figure 5: Gating strategies**

**A)** Gating strategies for the CD8^+^ (I) and CD4^+^ (II) T-cell populations shown in **Fig. 2**. in the manuscript **B)** Gating strategies for the B-cell population (IV) shown in **Fig. 2.** and alveolar macrophages (III) shown in **Fig. 4. C)** Gating strategies for the PMN (CD11b^+^Ly6G^high^, V), monocyte-like cells (Mo-like) (CD11b^+^Ly6C^high^CD11c^low^, VI) and iM/DC (CD11b^+^Ly6C^int^CD11c^high^, VII) populations in the lungs.

|  |
| --- |
| **Supplementary figure 6: mRNA expression levels of type 1 interferon-inducible genes in the lungs of Mtb-infected mice**  mRNA expression levels relative to *Gapdh* in the lungs of mice infected with Beijing-1585 (black dots), EAI-1627 (open circles) or H37Rv (grey squares). The combined expression of MX1, IFI44 and CCL2 into a Type 1 interferon signature in shown in **Figure 7C** of the manuscript. N=6 mice per group per time point, * p < 0.05, ** p < 0.01, *** p < 0.001, **** p < 0.0001 after Bonferroni correction |

**Supplementary data references:**

1 Huizinga, R. *et al.* Sialylation of Campylobacter jejuni lipo-oligosaccharides: impact on phagocytosis and cytokine production in mice. *PLoS One* **7**, e34416, doi:10.1371/journal.pone.0034416 (2012).

2 Rubio, D. *et al.* Crosstalk between the type 1 interferon and nuclear factor kappa B pathways confers resistance to a lethal virus infection. *Cell Host Microbe* **13**, 701-710 (2013).

3 Mourik, B. C. *et al.* Immunotherapy Added to Antibiotic Treatment Reduces Relapse of Disease in a Mouse Model of Tuberculosis. *Am J Respir Cell Mol Biol* **56**, 233-241 (2017).
